# Ethics at the Intersection of Intelligent Assistive Technology, Ageing, and the Home Environment: A Scoping Review

**DOI:** 10.1007/s11673-025-10479-8

**Published:** 2025-10-06

**Authors:** Elisabeth Langmann, Hans-Jörg-Ehni

**Affiliations:** 1 Institute of Ethics and History of Health in Society, Faculty of Medicine, University of Augsburg, Universitätsstr.2, 86159 Augsburg, Germany; 2 Institute of Ethics and History of Medicine, University of Tübingen, Gartenstraße 47, 72074 Tübingen, Germany

**Keywords:** Ethics, Healthcare, Ageing at home, Assistive technologies, Framework, Literature review

## Abstract

Intelligent assistive technology (IAT) is being developed to enable safe and autonomous ageing at home and is associated with supporting quality of life. These anticipated benefits must be balanced against potential unintended negative effects. This scoping review aims to identify and summarize the key ethical dimensions and frameworks discussed in the relevant scholarly literature. Furthermore, we examine whether the ethical dimensions identified can be derived from the four principles of bioethics, suggesting that, when properly specified, principlism could serve as a systematic framework for evaluating IAT. Thus, our review has two aims: identifying the ethical dimensions and frameworks currently discussed and investigating whether these frameworks can be structured according to the four principles or if additional principles are necessary. A systematic search across the databases PubMed, Web of Science, EMBASE, Belit, and PhilPapers, plus a manual search identified 535 publications, from which twenty-three studies were included. The results show twenty-one heterogeneous ethical dimensions, with similar matters considered across different categories. The review shows that key expectations and promises of developing and using IAT in this context mostly relate to the possibility of safely ageing at home and reducing healthcare costs. While assigning these ethical dimensions to the four principles of bioethics, it became clear that although all aspects discussed could be subcategorized, some ethical concerns might lose visibility or be inadequately addressed. We, therefore, conclude that the four principles generally provide a sufficient basis for evaluating these technologies.

**Supplementary Information:**

The online version contains supplementary material available at 10.1007/s11673-025-10479-8.

## Background

Our societies and the world we live in continue to be transformed by the rapid advancement of digital health technologies. These developments are expected to bring about unprecedented opportunities, especially through positively influencing processes of age-related changes (Neves and Vetere 2019). One significant development is the integration of artificial intelligence (AI) with existing assistive technologies, resulting in intelligent assistive technology (IAT) (Ienca et al. 2017). These systems aim to provide intelligent assistance and support to individuals with diverse needs by offering customized and adaptable solutions as effective tools to enable safe and autonomous ageing in the home environment (Cantone et al. 2023; Wangmo et al. 2019). Examples of IAT cover a broad spectrum and are designed to support and empower people living with disabilities. This includes sensor-based monitoring, wearables (such as smartwatches and GPS trackers), and devices (such as tablets or smartphones) with health-related applications. App-based IAT solutions promise to promote social engagement and health while prolonging independent living at home. Empirical evidence supports positive health and safety outcomes, such as a reduction in falls or appropriate medication use, as well as a reduction in concerns about unmet needs in emergencies (Lachal et al. 2016; Marikyan et al. 2019; Dratsiou et al. 2022). IAT also includes fall detectors and mobility aids, such as smart canes or wheelchairs with built-in sensors. In addition, voice-activated assistants (such as Alexa), distributed systems including smart homes with built-in sensors for light and heat, and situation-specific robots designed for tasks such as cleaning, soothing, and emotional response are considered to be IAT (Ienca et al. 2017). As these devices are designed for assistance, their potential to promote activities of daily living is closely related to their overall support of quality of life (QoL) (Turjamaa et al. 2019).

However, balancing the potential benefits of this (anticipated) transformed way of ageing against its risks and unintended side effects raises a plethora of questions at the intersection of digital technologies, well-being, ageing, and the home environment. As IAT promises to provide support in carrying out daily activities and healthcare, and, thus, in ageing well, specified objectives of such technologies and possible unintended side effects can be manifold (Krick et al. 2019; Tian et al. 2024). Hence, developing, implementing, and utilizing IATs require an ethical framework that can help identify and resolve ethical challenges, for instance by guiding the appropriate design of upcoming technologies or investigating how problems can be avoided during their usage. A recent editorial has emphasized the need to explore how digitalization and ageing intersect, highlighting the ethical tensions between autonomy, empowerment, and vulnerability in digital health solutions for older adults. It raises important concerns about what *ageing well* could mean in the context of digital health, whether technology design reinforces ageism, and how access to digital healthcare can be distributed equitably (Müller et al. 2024). Also, a recent systematic review has found that currently ethical considerations are not sufficiently considered in this context (Tian et al. 2024). Therefore, it is timely to provide an overview of the existing ethical discussions and frameworks, and suggest a possible structure for these which may be applied in this context.

Previous reviews discussing ethics in this context have focused primarily on aspects of privacy, security, autonomy, and justice (Demiris et al. 2009; Hofmann 2013; Gochoo et al. 2021; Felber et al. 2023). In addition, some articles from the field of research into technology acceptance investigate motives for the use and disuse of IAT (Peek et al. 2014; Turjamaa et al. 2019; Tsertsidis et al. 2019; Özsungur 2022; Wangmo et al. 2019). Among those studies, central ethical aspects, such as data misuse, stigmatization, the desire to live at home, or having control over technology, are also considered, but mostly without a direct reference to ethics (Peek et al. 2014; Zander et al. 2020). While discussions on the use of IAT by older adults living at home have increased, they remain fragmented, with key areas such as autonomy, privacy, and justice requiring further exploration. Thus, this scoping review aims to identify the main ethical aspects regarding the use of IAT in older age in the home environment. Our central objective is to provide an overview of the main ethical dimensions and frameworks discussed in the scientific literature itself and question whether they need to be adapted or expanded to map the ethical dimensions in the best way possible. With this, we intend to offer guidance for future research and practical work on developing and using technologies for older adults who age at home. In this regard, the present contribution is embedded in the multidisciplinary research project SMART-AGE, in which we aim to empirically test combined and interconnected IAT for improving the QoL of older adults living at home.

## Methods

Due to the exploratory nature of the research, a scoping review was chosen as the method to examine peer-reviewed literature that discusses the ethical dimensions of IAT for older adults living at home. According to (Tricco et al. 2018) guidance on this research approach, we decided to carry out a scoping review to (i) identify the extent of the literature available, (ii) clarify central ethical dimensions mentioned in the publications, (iii) recognize key characteristics related to the field of research, and (iv) analyse the state of the research and identify knowledge gaps. Conducting a scoping review appears to be the most appropriate approach, especially considering our main interest in identifying the ethical dimensions and frameworks used in the literature. The following steps were taken while performing the review: 1) defining the research questions mentioned above, 2) specifying eligibility criteria, 3) identifying relevant studies, 4) collecting the data, 5) compiling and summarizing the data, as well as 6) reporting and interpreting the results.

### Eligibility Criteria

The following eligibility criteria were defined for individual publications to be included in the review: (i) peer-reviewed publications discussing ethical dimensions related to (ii) welfare technology, gerontechnology, smart houses, assisted living, and assisted housing; (iii) concerning older adults, and (iv) published in German or English. Studies were excluded when (i) not explicitly discussing ethical issues, (ii) not focusing on older adults as a population, and (iii) being published in the wrong type of publication (e.g., nonscientific journals, as editorial letters, posters, or dissertations), (iv) only exploring a specific sub-topic (e.g., focusing on a specific disease, such as dementia), (v) or concentrating exclusively on robots. The decision for exclusion criteria (iv) and (v) was based on the following considerations: Firstly, we did not aim to focus on a specific health condition. Secondly, there are a relatively large number of contributions on robots, which indicates that this technology has its own risks and requires a separate and more specific ethical evaluation. We, therefore, decided to exclude robots and the discussion of the ethical challenges they may create. In addition, no specific definition of such technologies was used to display the full spectrum of ethical dimensions and frameworks used in the literature.

### Search Strategy

After defining the research objective and eligibility criteria, the following search strategy was conducted: The databases PubMed, WebofScience, EMBASE, Belit, and PhilPapers were systematically searched in February 2024 using a combination of the following five keyword categories and associated terms in each category: (1) “older people” and “elderly,” (2) “home care” and “aging at home,” (3) “telecare” and “gerontechnology,” (4) “health,” and (5) a variety of terms were used for keywords related to ethical aspects to cover a wide range of publications: “ethics,” “quality of life,” “ageing well,” and “social interaction.” Table 1 in the appendix presents a full list of all the search terms used and an exemplary search string for WebofScience. We considered a variety of types of studies, including empirical and argument-based publications as well as reviews. Additionally, we performed a manual search of relevant journals and platforms dedicated to specific themes to find additional suitable literature.

### Screening and Selection of the Studies

**Fig. 1:**
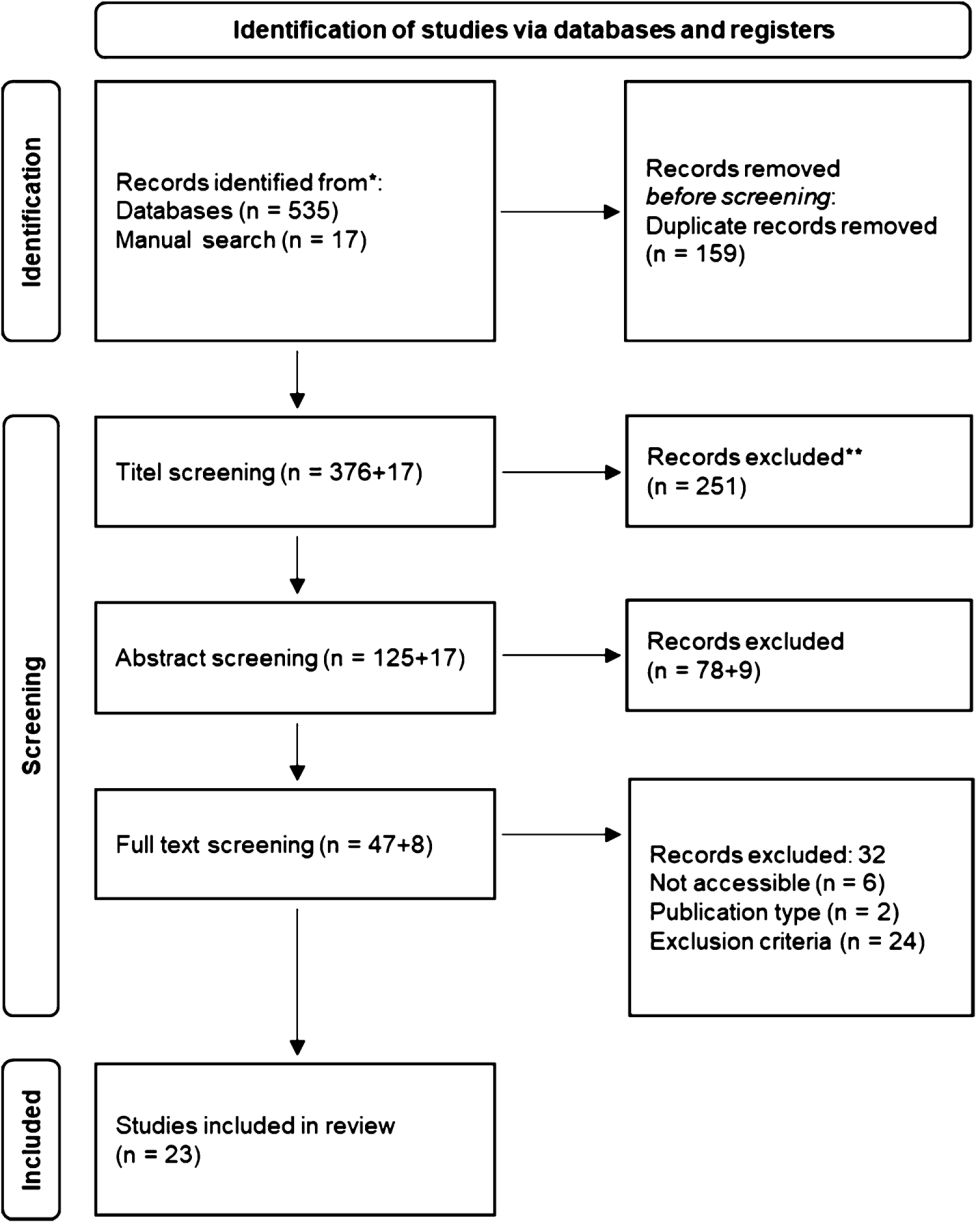
PRISMA: Selection of the studies

During the study selection process, we adhered to the PRISMA guidelines (Tricco et al. 2018) and followed the criteria outlined in Fig. 1. The author EL conducted the screening procedure independently. When uncertainties occurred, the author HJE was consulted to reach an agreement. We identified 535 publications through database searches using the specified keywords as well as by means of manual searches. After removing duplicates, we reviewed the titles and abstracts based on the predefined eligibility criteria to determine which studies to include or exclude, resulting in fifty-five articles for full-text review. Following this detailed assessment, twenty-three studies met the inclusion criteria and were incorporated into the final analysis. All studies selected were published in English and ranged from 2011 to 2023 in terms of publication dates. The articles selected varied in type, offering a diverse perspective on the topic. The articles reviewed are very heterogeneous in terms of the type of publication, including different types of reviews (n = 9) (Sánchez et al. 2017; Zhu et al. 2021; Zwijsen et al. 2011; Sundgren et al. 2020; Moraitou et al. 2017; Chung et al. 2016; Pirzada et al. 2022; Felber et al. 2023; Ji and Kim 2022), normative analyses (n = 7) (Schicktanz and Schweda 2021; McLean 2011; Bennett 2019; Panico et al. 2020; Sonnauer and Frewer 2023; Rubeis 2020; Rubeis et al. 2022), empirical studies (n = 5) (Ehrari et al. 2020; Mortenson et al. 2016; Birchley et al. 2017; Sánchez et al. 2019; Ienca et al. 2021), qualitative literature analyses (n = 1) (Hartmann et al. 2023) and horizon scanning (n = 1) (Flick et al. 2020). Based on the decision to include reviews, the number of results considered indirectly within the analysis presented increased considerably. This strengthens the significance of the ethical aspects discussed in this literature review.

### Data Extraction and Qualitative Synthesis

After identifying the relevant literature, the data were qualitatively and systematically extracted from the publications included. Accordingly, we drafted a form in Microsoft Excel that contains an overview of the ethical dimensions of each publication. The findings of each of the ethical dimensions, as explicitly stated in the eligible literature, were categorized within our Excel sheet and presented descriptively. The resulting allocation to individual ethical dimensions was based on the categorizations listed in the respective publications. Furthermore, an additional content analysis was performed to systematically reduce, synthesize, and structure the data to extract and categorize the ethical aspects reported in a more precise manner and to evaluate the variety of dimensions highlighted in the literature in accordance with principlism.

## Results

**Fig. 2:**
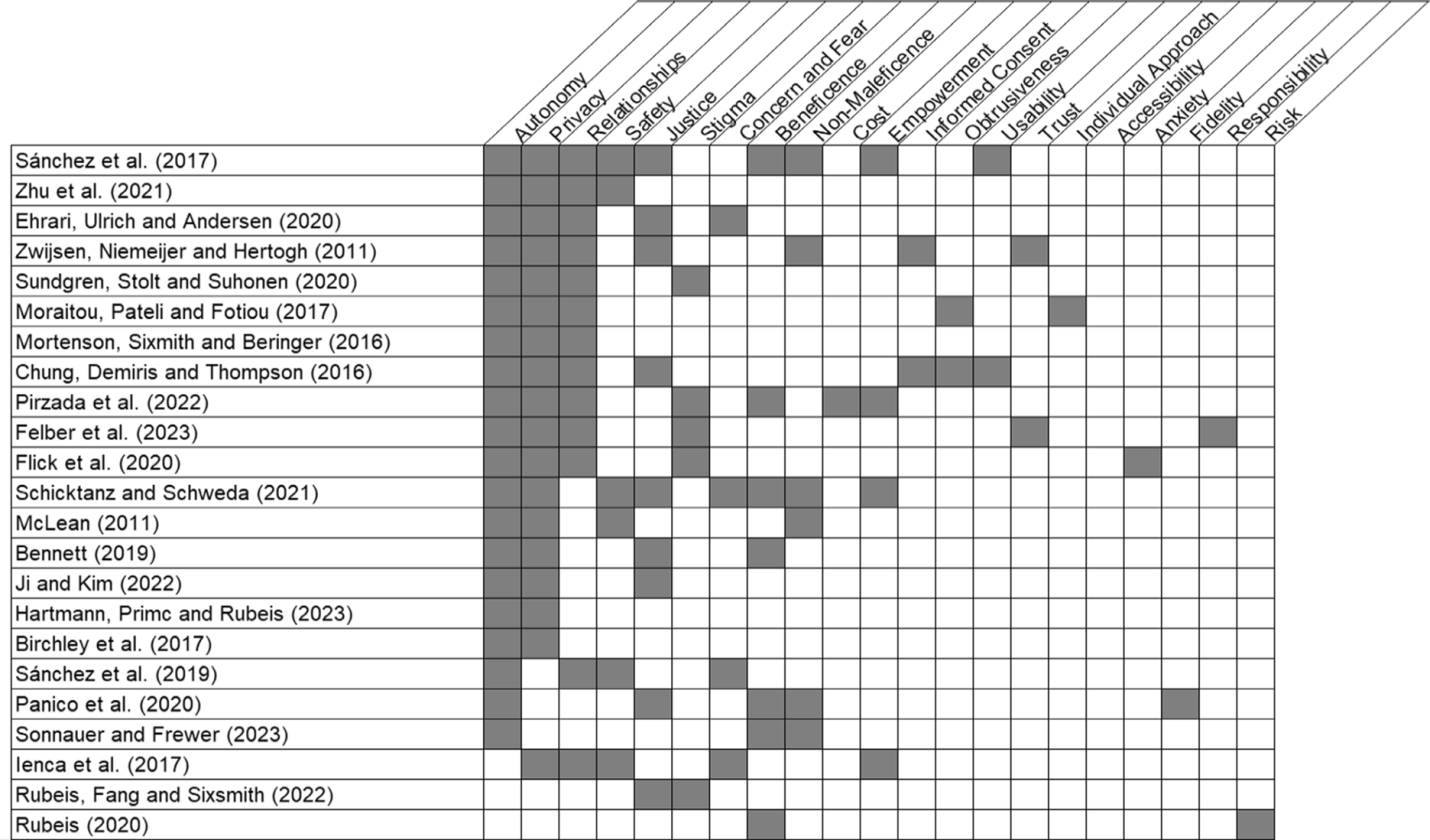
Ethical dimensions pointed out in the literature

Our analysis revealed twenty-one different ethical dimensions pointed out in the eligible publications. As can be seen in Fig. 2, the respective ethical dimensions were mentioned at different frequencies and in varying combinations, resulting in *autonomy, privacy*, *relationships*, and *safety* being the four most frequently stated. The category of *relationships* was discussed under many different terms, such as face-to-face communication, human contact, social participation, and social network (Flick et al. 2020; Sundgren et al. 2020; Chung et al. 2016; Zwijsen et al. 2011; Zhu et al. 2021; Ienca et al. 2021). The overarching designation *relationships* was chosen by the authors of this contribution to combine all those aspects. Interestingly, only three of the twenty-three publications reviewed specifically utilized distinct frameworks to examine ethical issues in the context of IAT in the home environment. These frameworks included Hofmann’s questionnaire (n = 1) (Sánchez et al. 2017), principlism as a critical point of departure (n = 1) (Schicktanz and Schweda 2021), and the “4-d risks” model (n = 1) (Rubeis 2020). The other twenty publications used various approaches to discuss ethical considerations. This finding was both surprising and expected for several reasons. On the one hand, the limited use of the widely known four principles—autonomy, beneficence, nonmaleficence, and justice—defined by Beauchamp and Childress (2019) was unexpected, given their prominence in ethical discussions. On the other hand, previous studies had already identified privacy and safety as significant ethical concerns when it comes to IAT, especially among general users and older adults (Gochoo et al. 2021; Birchley et al. 2017; Zander et al. 2020). Thus, the lack of a dominant ethical framework aligns with the broader focus on these specific issues. Additionally, the literature reviewed often addressed various technologies without distinguishing them in detail, making it challenging to draw specific conclusions about the ethical implications of different types of technology. This generalization points to a need for more nuanced analyses to understand how different IAT devices or systems might raise unique ethical concerns.

In order to achieve the objective of this contribution, we grouped the ethical aspects identified in accordance with the four principles of bioethics developed by Beauchamp and Childress with the aim of questioning their applicability for systematic evaluations in this context. Similar aspects were summarized in subcategories (see Table 2 in the appendix). The ethical aspects are presented concerning the four principles plus specific trade-offs and discussed thereafter in the following sections.

### Respect for Autonomy

*Autonomy* is a major topic in the discourse on the use of digital technology in the home environment and is accordingly pointed out as such in twenty of the articles included (see Fig. 2). It is discussed as a priority and central ethical dimension in almost all articles analyzed. The usage of different types of technologies is frequently promoted to maintain autonomy and QoL for older adults (Zwijsen et al. 2011; Felber et al. 2023). This refers to an increase of autonomy understood as independence or agency, which we will discuss under potential benefits of these technologies (see beneficence below). It is crucial to clearly distinguish autonomy as self-determination or free choice in the use of IAT from these benefits. Respecting autonomy—ensuring individuals can make free and informed choices—is distinct from the potential benefit of increased independence, as independence from external support does not necessarily guarantee autonomous decision-making. Thus, grouping passages that concern the respect for autonomy, as understood and outlined by Beauchamp and Childress (Beauchamp and Childress 2019), *decision*, *control*, *privacy*, and *interdependence* emerge as pivotal facets of autonomy within the scope of the analysis.

Thereby, *decision* addresses both restricting and enhancing factors concerning autonomy that are mainly associated with decision-making in different contexts, such as the willingness to use certain technologies (Sánchez et al. 2017; Zhu et al. 2021) and the right to accept or reject them. It encompasses the capacity and right to make autonomous choices without undue influences (Schicktanz and Schweda 2021; Panico et al. 2020; Ehrari et al. 2020). Additionally, the question of who ultimately decides on the adoption of IAT, considering the complex family dynamics involved (McLean 2011), is crucial. To avoid fostering dependence it is important that decisions should be tailored to individual needs, not merely driven by technology availability (Zwijsen et al. 2011).

In this context, *informed consent* is pointed out as central for ensuring autonomy and, thus, as a crucial instrument for overcoming ethical challenges related to choice and privacy (Zwijsen et al. 2011; Sánchez et al. 2017). Difficulties regarding informed consent itself are identified in the presentation and provision of information; striking a balance between sufficient detail and avoiding overwhelming complexity, especially for those without prior technological knowledge ((Zwijsen et al. 2011; Zhu et al. 2021). One study found that older users are often more concerned with the purpose of technology than details about functionality, also regarding sensitive topics, such as data privacy (Chung et al. 2016). Hence, informed consent represents a sensitive area that requires thoughtful planning and design and should be regarded as an ongoing process (Sánchez et al. 2017; Hartmann et al. 2023).

Furthermore, *control* over IAT is a paramount aspect of autonomy that comes up in the eligible literature. Especially in the papers by Sundgren et al. 2020 and Pirzada et al. 2022, having control over technology is discussed as a sense of independence, at the same time, raising the issue of who would control the technology in case the affected person’s capacity to do so declines (Sánchez et al. 2019). Negative effects, such as potential risks for autonomous decision-making, are also debated. These include family or caregivers pressuring older adults to use technology (Sundgren et al. 2020; Zwijsen et al. 2011; Felber et al. 2023), often to ease the former’s own minds (Sundgren et al. 2020; Zwijsen et al. 2011; Sánchez et al. 2017) interpret this as a risk for older adults to become passive regarding the choice to use technologies for daily support, which could lead to a misinterpretation of acceptance. Additionally, pressure to use IAT can be linked to feelings of frailty and dependence in older adults (Pirzada et al. 2022). Moreover, this pressure can be based on a tendency towards overprotection and paternalistic benevolence by family and formal caregivers (Sánchez et al. 2019; Sundgren et al. 2020). In turn, the fear of becoming a burden when older is discussed as a motive to maintain autonomy through the use of technology (Sánchez et al. 2019; Sundgren et al. 2020). Conversely, technology dependence is outlined as a major topic concerning the possible negative effects of technology use on autonomy. Thereby, becoming overreliant on assistance through technology and, consequently, creating functional dependencies is described as a central concern of losing autonomy through the use of technology (Sánchez et al. 2017; Sánchez et al. 2019; Chung et al. 2016; Flick et al. 2020; Zwijsen et al. 2011; Moraitou et al. 2017). As a result, pressure and aspects of being a burden are interconnected and are central facets of the context of ageing and using IAT. In this regard, a relational understanding of autonomy is discussed (McLean 2011; Sonnauer and Frewer 2023). It emphasizes that needing help should not be interpreted as a lack of autonomy (McLean 2011) and that self-determination can coexist with dependence on external support (Hartmann et al. 2023). This challenges traditional views of independence, recognizing that autonomy extends beyond individual capabilities and encompasses social determinants and individual diversity (Hartmann et al. 2023).

As a subcategory of autonomy, *privacy* is discussed as an ethical dimension in eighteen of the articles included (see Fig. 2). It encompasses multiple aspects, including personal space, freedom from observation, and respect for autonomy (Zwijsen et al. 2011). Various studies suggest that remaining at home can offer greater privacy compared to residential aged care facilities (Sundgren et al. 2020; Ehrari et al. 2020; Felber et al. 2023; Ji and Kim 2022), with IAT often facilitating this independence and privacy (Sánchez et al. 2017). However, what is a growing concern is the pressure to accept data tracking in exchange for independent living (Hartmann et al. 2023). The privacy discourse extends to ethical considerations surrounding data collection, storage, sharing, and protection (McLean 2011; Birchley et al. 2017). This is further emphasized by the prominence of concerns regarding data access and privacy (Chung et al. 2016; Hartmann et al. 2023; Ehrari et al. 2020; Ji and Kim 2022; Zhu et al. 2021). While technological advancements offer new possibilities for monitoring and assistance, there is a delicate balance to strike between autonomy and privacy protection (Ehrari et al. 2020; Ji and Kim 2022; Bennett 2019). This balance is further complicated by the varying emphases on informational (e.g., data protection) and physical privacy (e.g., avoiding intrusive monitoring) (Birchley et al. 2017). Ultimately, the challenge lies in ensuring that technological innovations uphold individual autonomy while respecting the right to privacy.

### Beneficence

Beneficence is a key ethical dimension in the discourse on IAT and is discussed in fourteen of the twenty-three articles included (see Fig. 2). Based on the literature analysed, the outlined benefits of using diverse IAT can be subsumed under *safety*, *empowerment*, and *relationships*, and result mainly from a combination of aspects that promote QoL when ageing at home (Schicktanz and Schweda 2021). *Safety* is primarily assessed as being safe in emergency situations when living at home independently (Moraitou et al. 2017; Schicktanz and Schweda 2021) and getting help and support when needed (Mortenson et al. 2016). Ageing and health-related emergencies are discussed in several of the articles reviewed in terms of being prepared *through* the use and support of certain technologies (Mortenson et al. 2016). Thereby, it has become evident that the promise of being safe at home is the main motivation for older adults to use IAT. It is also described that some older adults consider surveillance options by certain technologies to be beneficial in making them feel safer. This is specifically related to providing some relief to not only caregivers but also the users themselves when living at home independently (Panico et al. 2020; Mortenson et al. 2016; Zwijsen et al. 2011; Pirzada et al. 2022). Informal caregivers especially appear to expect and perceive benefits, such as emotional relief, more strongly than older adults for whom the technology is intended (Sundgren et al. 2020; Zhu et al. 2021).

In addition to the perceived safety benefits offered by the use of such technologies, it is also plausible to ascertain benefits in terms of *empowerment*. This is primarily reflected in the expected benefit of independence in older age (Pirzada et al. 2022). It is linked not only to increase self-esteem and self-confidence (Sundgren et al. 2020) but also, quite fundamentally, through promoting independent living. In this context, supporting autonomy is understood as living with less support from family or professional caregivers. The use of these technologies can reshape existing power dynamics, as evidenced by the factors discussed above (Schicktanz and Schweda 2021; Pirzada et al. 2022; Sánchez et al. 2019; Mortenson et al. 2016; Zhu et al. 2021; Ienca et al. 2017). Therefore, empowerment in concordance with self-determination through living at home (Schicktanz and Schweda 2021; Zwijsen et al. 2011) and independence from family and caregivers (Sánchez et al. 2019; Zhu et al. 2021) can be highlighted as one of the main benefits of such technological advancement. Additionally, these empowerment effects have been documented as relieving emotional tension and reducing burdens associated with the care of ageing relatives in families and care by informal caregivers ((Sundgren et al. 2020; Zhu et al. 2021; Pirzada et al. 2022). Thus, a notion of empowerment that reduces reliance on other people is expressed (Zhu et al. 2021) but includes independence through the assistance of technology in daily tasks and the use of help during emergencies (Sundgren et al. 2020; Mortenson et al. 2016). As a consequence of this kind of empowerment, aspects of privacy, such as a strong sense of privacy while using IAT in contrast to receiving care from relatives (Sánchez et al. 2017) and an increased sense of privacy compared to moving into a residential aged care facility (Sundgren et al. 2020), can also be understood as important benefits of IAT in the context at hand. While these aspects already concern social interactions, *relationships* are the third ethical dimension most mentioned in the articles included (n = 13) and, thus, a central subtopic of beneficence affected by IAT for older adults in the home environment. The literature indicates that digital technologies can enhance social interactions in a variety of ways, e.g., video-calling to maintain or even increase *relationships* (Zhu et al. 2021; Zwijsen et al. 2011). Some results show that, through technologies, older people express a desire for support and even empowerment in digital and face-to-face communication (Chung et al. 2016).

Accordingly, the literature demonstrates that IAT holds significant promise for enhancing the well-being of older adults who desire to age in place. These benefits are connected primarily with empowering individuals to manage their own health (Ehrari et al. 2020), fostering a greater sense of well-being through supporting ageing at home, and providing comfort to caregivers through remote monitoring capabilities (Bennett 2019).

### Non-Maleficence

The ethical aspects of *non-maleficence* are discussed in six of the twenty-three articles included (see Fig. 2). These discussions focus on whether and how IAT could cause harm to users or to the extent to which users might be exposed to harm when using these technologies. *Safety*, *privacy, relationships,* aspects concerning *stigma*, and *sustainability* can be derived as subtopics.

As mentioned in the context of *beneficence,* while IATs can increase safety for independent living, their use also raises potential concerns. The proclaimed *safety*, for example, could be compromised when systems fail to detect emergency situations, such as falls (Sánchez et al. 2017). These risks often stem from insufficient robustness, leading to ineffective equipment and liability issues. Additionally, it is important to note that technical errors and malfunctions, such as inaccurate measurements, can pose significant harm (Ehrari et al. 2020; Schicktanz and Schweda 2021; Panico et al. 2020; Moraitou et al. 2017; Sánchez et al. 2017). This is particularly important given the widespread safety claims associated with these technologies.

In addition, privacy issues related to the use of IAT in the home environment have been identified as a highly sensitive issue, particularly regarding the various risks of privacy violations and the resulting potential harm, and are discussed in eighteen of the twenty-three articles included (see Fig. 2). Three main sources of privacy violations can be identified in this context: (1) misuse of data by obtaining or sharing information without permission and the abuse of information, e.g., by insurance companies (Ehrari et al. 2020; Zhu et al. 2021; Chung et al. 2016; Ienca et al. 2017; Felber et al. 2023; Flick et al. 2020). (2) Surveillance, mostly through using cameras in the home environment, is interpreted as fundamentally intrusive and a major risk to privacy (Hartmann et al. 2023; Sonnauer and Frewer 2023; Ienca et al. 2017; Zwijsen et al. 2011; Pirzada et al. 2022). Furthermore, it is noted that surveillance can also lead to self-surveillance, which has been discussed as a potential concern due to its implications for autonomy and privacy (Hartmann et al. 2023; Mortenson et al. 2016; Rubeis 2020). (3) Obtrusiveness of technologies, which is often associated with their location (e.g., in the bedroom or bathroom) or size (Chung et al. 2016; Felber et al. 2023; Bennett 2019). Again, this is particularly highlighted in connection with cameras, which were predominantly considered as critical regarding privacy concerns and interpreted as a major factor for triggering anxieties and provoking unease (Sánchez et al. 2019; Chung et al. 2016; Bennett 2019). Thus, such technologies can, among other things, stimulate psychological stress and cause unintended, adverse outcomes. Cameras are particularly rated as very critical to privacy throughout the literature.

Despite privacy being a central concern in the utilization of IAT, it can be observed that, as health limitations increase, individuals are more willing to make trade-offs to maintain their independence and safety at home (Ienca et al. 2017; Mortenson et al. 2016; Chung et al. 2016; Sánchez et al. 2017; Sundgren et al. 2020; Pirzada et al. 2022; Felber et al. 2023; Hartmann et al. 2023). However, while there is a growing acceptance of sacrificing privacy for the benefits of ageing in place with technological support, the literature also presents a variety of associated concerns and fears. Central to this are concerns regarding the loss of a feeling of the home as a private space and the fear of being watched and controlled by someone known or unknown (Zwijsen et al. 2011; Sánchez et al. 2019; Mortenson et al. 2016; Sundgren et al. 2020).

Moreover, the literature analysed suggests that certain IAT could negatively impact aspects of personal *relationships*. Many articles, for instance, point out the concern of a potential decrease in in-person interaction due to IATs that offer alternative communication methods, such as video calls (Ienca et al. 2017; Zwijsen et al. 2011; Zhu et al. 2021; Moraitou et al. 2017; Sundgren et al. 2020; Flick et al. 2020). This could weaken family bonds and potentially impact healthcare delivery if healthcare visits shift to a predominant online mode (Sánchez et al. 2017). Hence, concerns exist that telecare may unintentionally discourage social interaction, reduce mobility, and negatively impact overall well-being. By enabling older adults to stay home more, telecare could contribute to feelings of isolation and potential health problems (Sundgren et al. 2020). Moreover, an overreliance on technology for safety might create a fear of going outside, further exacerbating feelings of isolation (Pirzada et al. 2022). These trends raise significant ethical concerns regarding the balance between technological advancement and preserving human connection and autonomy. Moreover, the overreliance on IAT has been associated with adverse effects on users’ well-being (Sánchez et al. 2019; Sánchez et al. 2017; Chung et al. 2016; Flick et al. 2020; Zwijsen et al. 2011; Moraitou et al. 2017; Schicktanz and Schweda 2021). Additionally, Rubeis argues that the widespread adoption of such technologies in nursing may negatively impact patient–nurse relationships by potentially depersonalizing older adults and limiting nurses’ ability to integrate individual patient experiences into the clinical processes (Rubeis 2020).

In addition to the potential adverse effects on face-to-face interaction, overlooking the importance of direct contact in health-related data collection can have significant consequences. This oversight may accentuate disabilities and deficits, potentially leading to the disregard of holistic notions of individuals. Such concerns are closely linked to the fear of individuals being reduced to mere data points rather than being treated as real-life people (Sundgren et al. 2020; Sánchez et al. 2017). In addition, Rubeis examines the concept of the datafication of older adults in this context, suggesting that AI-based gerontechnologies may contribute to their dehumanization. By prioritizing quantifiable clinical data, such as vital functions or behavioural patterns, these technologies may result in the marginalization of individual experiences and narratives, ultimately limiting the holistic comprehension of an older person’s condition (Rubeis 2020).

Furthermore, certain aspects of *stigma* can be assigned to *non-maleficence*. This includes a deficit-oriented perspective on older people who use IAT, as well as prejudice and stereotypes concerning older age. The articles analysed suggest that this stigma harms users by reinforcing a deficit-oriented perspective, emphasizing older adults’ limitations and disabilities, and turning IAT into a divisive tool, differentiating between those who require support and those considered “normal” (Sánchez et al. 2017; Mortenson et al. 2016). Consequently, using IAT becomes a marker of frailty and dependence, leading to stigmatization (Zwijsen et al. 2011; Felber et al. 2023). What results from this perception is that it discourages IAT adoption among older adults and raises concerns about data privacy and potential judgment based on sensor data (Chung et al. 2016; Pirzada et al. 2022). Research suggests that older adults may fear being judged for needing help with technology, leading to feelings of incompetence and shame (Flick et al. 2020; Sundgren et al. 2020; Felber et al. 2023; Ehrari et al. 2020), which can manifest as a fear of being seen as being reliant on technology or disabled in public (Sánchez et al. 2019; Flick et al. 2020). This concern aligns with Felber et al.’s findings on how ageism and stigma influence older adults’ preferences for discreet technologies and their openness to acknowledging a need for assistance (Felber et al. 2023). Thus, it is not the technology itself that creates negative effects but rather the anticipated societal reactions to using assistive products. Furthermore, biases among developers can perpetuate prejudices, contributing to exclusionary practices (Flick et al. 2020). Rubeis warns that ageist stereotypes may become deeply embedded in the design frameworks of such technologies, thereby perpetuating discrimination and marginalization (Rubeis 2020). One notable manifestation of ageism in IAT is the concept of age scripts, where specific views on ageing are incorporated into the design, resulting in the imposition of simplistic notions of older age onto users. As users interact with these technologies, they are compelled to adapt to these predefined scripts, limiting their autonomy and reinforcing ageist stereotypes (Rubeis 2020; Rubeis et al. 2022). In order to address these issues, Rubeis et al. advocate for equity-focused approaches in selecting training data and designing technologies for older adults. Integrating social determinants into AI-based AgeTech and adopting an intersectional approach are, thereby, understood as crucial to mitigating discrimination and digital marginalization (Rubeis 2020).

Notwithstanding the above, Schicktanz and Schweda propose that *sustainability*, particularly concerning low waste and renewable energy, should be a central ethical aspect of developing and using these technologies (Schicktanz and Schweda 2021). They highlight sustainability as a central aspect of justice, but our analysis suggests it can also be aligned with the principle of *non-maleficence*. This perspective on sustainability emphasizes the potential environmental damage these technologies may cause and the need to conserve limited resources. Overall, incorporating these diverse aspects into the principle of non-maleficence allows for a comprehensive understanding of efforts needed to avoid harm through IAT.

### Justice

*Justice* is explicitly discussed in nine of the twenty-three articles included (see Fig. 2). It is primarily understood in the literature selected as respecting the rights of users as well as ensuring equal and fair access to the use of IAT, while also taking into account the related costs (Panico et al. 2020; Chung et al. 2016; Schicktanz and Schweda 2021; Sánchez et al. 2017; Zhu et al. 2021). Consequently, *costs* and *accessibility* are central dimensions discussed in the literature regarding justice. Accordingly, by consolidating the respective overlapping categories, a structured overview of the ethical aspects of justice related to the use of technology in older age emerges.

In general, IAT is considered a promising and (mostly) cost-effective way for healthcare systems to provide and dispense care to people needing to access those services (Schicktanz and Schweda 2021; Sánchez et al. 2017; Zhu et al. 2021; Panico et al. 2020). In this context, facets of usability and different kinds of barriers to the use of IAT, for example, language barriers, are discussed (Chung et al. 2016; Zhu et al. 2021; Rubeis et al. 2022; Ji and Kim 2022). In addition, these technologies are subject to other barriers in the context of justice, especially depending on who covers the costs (Zhu et al. 2021; Chung et al. 2016; Sánchez et al. 2017; Rubeis et al. 2022; Ji and Kim 2022; Bennett 2019). Based on the literature analysis, it can be concluded that if the respective technologies have to be paid for by individual users, there is a risk that only those with sufficient financial means will be able to employ them (Pirzada et al. 2022; McLean 2011; Bennett 2019), with the result of exacerbating existing inequalities and vulnerabilities. The articles included also indicate that acceptance and usage increase if family members or insurance cover the costs of the respective technologies. Rubeis et al. highlight that AI-based AgeTech may not fit the needs and resources of users in low-income countries; in order to reduce access barriers, a well-informed design process should include user experiences from various groups, and the context of technology design should be transparent (Rubeis et al. 2022). Both Pirzada et al. and Rubeis et al. conclude that technologies that support care should be made less expensive or subsidized for a more inclusive society (Pirzada et al. 2022; Rubeis et al. 2022).

## Discussion

The findings of this scoping review highlight the complexity and multifaceted nature of ethical dimensions associated with IAT in the context of ageing at home. The identification of twenty-one distinct ethical dimensions within the literature underscores the diversity and heterogeneity of the concerns and considerations that researchers and practitioners must navigate. This diversity, while reflective of the broad scope of ethical issues, also poses challenges in developing a systemic and context-sensitive framework for ethical evaluation.

A significant insight from this analysis is the dominance of autonomy, privacy, relationships, and safety as the most frequently discussed ethical dimensions. These core aspects align closely with the principles of bioethics (Beauchamp and Childress 2019). Although principlism is not central in the reviewed publications, referencing it provides a practical way to categorize ethical dimensions by leveraging its familiarity. Referring to the question of whether an adaptation or expansion is needed to most effectively map the ethical aspects identified in the literature, we conclude that applying or referring to principlism is beneficial in terms of identifying and conceptualizing ethical issues as it provides a common language and guidance (Bosk 2010).

However, its broad categories may obscure critical concerns, such as the interplay between surveillance and autonomy. This points to the need for more context-sensitive approaches, such as relational autonomy or care ethics, which align with critiques in the broader digital health ethics literature (Perkins et al. 2012; L. Liu et al. 2022). This suggests a need for a more nuanced, context-sensitive ethical framework that incorporates key ethical dimensions beyond the four principles to address the unique challenges of IAT for older adults living at home.

The lack of structured ethical frameworks in the available literature reveals a critical gap in the systematic evaluation of IAT. While some studies reference frameworks such as Hofmann’s questionnaire, principlism, or the “4-d risks” model, the predominant approach remains ad hoc. This aligns with broader trends in digital health ethics, where issues like privacy, surveillance, and safety are prominent discussion points (Ienca et al. 2021; Grosman-Rimon and Wegier 2024). The lack of theoretical grounding raises important questions about how ethical priorities are determined in IAT research and development. Different studies underscore this gap by highlighting that many IATs for older adults are designed without explicit ethical considerations, which may hinder their effective implementation in real-world settings (Ienca et al. 2018; Wangmo et al. 2019). This fragmented approach poses challenges for systematic ethical evaluation and underscores the need for ethical frameworks specifically tailored to the complexities of IAT for older adults in home environments.

A particularly dominant theme in the literature is the tension between autonomy as a guiding ethical principle and the ways in which it is both enabled and constrained through technology. IAT is often framed as maintaining autonomy by supporting independent living and reducing reliance on caregivers (Zwijsen et al. 2011; Felber et al. 2023). However, as our analysis shows, autonomy in this context is highly contingent on external factors, such as the financial accessibility of IAT, the design of technologies, and the power dynamics between older adults, caregivers, and institutions (McLean 2011; Mortenson et al. 2016). This raises important questions concerning how autonomy is operationalized. Scholars such as Anita Ho (2023) argue that AI-driven health monitoring does not merely extend autonomy but reshapes it, creating new forms of dependency and vulnerability. Thus, IAT may place individuals in difficult positions where they feel pressured to adopt monitoring tools due to social expectations or systemic constraints, a concern echoed by other scholars (Lu 2024; Wangmo et al. 2019).

Moreover, the literature indicates that while informed consent is often emphasized as a safeguard for autonomy, it may not fully account for the complexities of decision-making in this context (Sánchez et al. 2017). As such, while IAT can offer users greater independence, it can also introduce dependencies and constraints that require deeper ethical reflection. This raises important questions about whether existing ethical frameworks sufficiently capture these complexities or if more nuanced approaches are necessary to account for the structural influences on decision-making in this space.

Beyond autonomy, the ethical dimensions categorized under beneficence highlight the anticipated advantages of IAT, including improved safety, social connectivity, and QoL (Pirzada et al. 2022; Panico et al. 2020). However, the literature also points to significant tensions. While these technologies are intended to enhance well-being, they may simultaneously introduce new vulnerabilities. Privacy concerns, for instance, highlight how users may accept data tracking as a means of increasing security, yet this can come at the cost of increased surveillance and a potential loss of personal control (Ienca et al. 2017; Felber et al. 2023). Similarly, technological dependency is a central concern: while IAT is often framed as fostering independence, its use may, in some cases, diminish human contact, raising concerns about social isolation (Zhu et al. 2021; Sundgren et al. 2020; Flick et al. 2020; Sánchez et al. 2017). These concerns underscore the need to carefully consider how technology is integrated into care environments. The principle of non-maleficence, therefore, should not only consider individual risks and benefits but also take into account broader societal implications, including the discussed potential dehumanization of care through algorithmic decision-making and the reinforcement of deficit-oriented narratives of ageing (Rubeis 2020; Hartmann et al. 2023). These findings contribute to ongoing debates about the risks of depersonalization (Topol 2019; Padalkar et al. 2024) and systemic bias in digital healthcare, such as discussions around ageism and its impact on the design and implementation of digital health technologies (Neiertz et al. 2023; Peine and Neven 2019; Mannheim et al. 2023).

Contributing to a more inclusive society also requires focusing on nondiscrimination as a central part of justice. Therefore, aspects subsumed under non-maleficence, such as potential stigmatization through the use of IAT, also need to be included and addressed in discussions about justice. Consequently, both the economic gap and facets of the digital divide between users need to be considered with the aim of using IAT to enhance access to healthcare and support the pursuit of distributive justice in health. However, the articles reviewed mainly discuss *justice* in light of economic issues and accessibility. A discussion of the social determinants of equality and crucial facets of nondiscrimination regarding the development, implementation, and use of IAT is lacking in the literature included. This is consistent with general problems in the application of digital technologies in the field of health, namely, that bias and the risk of discrimination against minority groups when using IAT also receive too little attention so far (Rubeis 2020). Furthermore, there is minimal mention of ageism, despite its relevance in this specific context. Therefore, future discussions should consider these issues more widely, preferably from an intersectional perspective.

## Strengths and Limitations

This literature review has notable strengths and limitations. We conducted a systematic search across multiple databases (PubMed, EMBASE, WebofScience, Belit, and PhilPapers) and supplemented it with a manual query. Due to duplicate records and narrow inclusion criteria, only a small number of articles met the selection criteria. While this ensured that the included studies were highly relevant, it may have excluded indirectly related data. A key strength of our approach is its systematic and transparent methodology, enhancing reproducibility. However, content analysis relies on the reviewers’ expertise in ethics, introducing a degree of subjectivity. To mitigate bias, we applied a structured categorization process.

The inclusion of heterogeneous studies with varying definitions, terminologies, and measurement tools presents a challenge, potentially leading to inconsistencies and limiting the generalizability of our findings. Consequently, the outcomes of this review may be relevant only to specific contexts or populations. The focus on particular settings limits the broader applicability of the findings across all IAT implementations. Additionally, while this review identifies and discusses ethical dimensions based on existing literature, it lacks empirical validation through primary research, which could provide more concrete evidence of these concerns. Future research should incorporate primary studies to substantiate the ethical concerns identified, and a questionnaire study being a promising next step.

## Conclusion and Outlook

To the best of our knowledge, this scoping literature review is the first to address the questions of key ethical dimensions and frameworks used in the context of IAT, ageing at home, and ethics. This review shows that the variety of twenty-one ethical dimensions pointed out in the literature can be subsumed under the four principles of bioethics. In this respect, we argue that using principlism is beneficial due to its familiarity and, accordingly, offers cross-disciplinary orientation in questions of ethics in this context. However, the application of these principles alone may not adequately capture all the nuanced ethical concerns specific to IAT, suggesting a need for more detailed and context-specific ethical frameworks.

In addition, this review highlights that IAT is often promoted with high expectations, particularly regarding ageing at home and healthcare cost reduction. However, empirical evidence on whether these expectations are realized remains limited. Addressing this gap requires evaluating the real-world impact of IAT beyond theoretical claims, with a focus on its implications for autonomy, justice, and potential benefits and harms. While justice is often discussed in terms of economic access, broader issues such as digital exclusion, bias in IAT, and marginalization of older adults remain underexplored. Future research should incorporate intersectional approaches to analyse how age, disability, socioeconomic status, and digital literacy intersect in shaping the impact of IAT.

Finally, the widespread ageism within our society (Chang et al. 2020) should be considered a likely influence in this context. As part of future research, it will be necessary to examine whether the age of potential users should be considered a crucial variable—not from a deficit-oriented perspective, but in light of how ageism shapes technology design, implementation, and adoption. Rather than focusing on chronological age, it seems particularly important to explore whether health-related needs are the decisive factor in the use of certain technologies. In this respect, engaging more closely with research in disability studies could provide important insights.

## Supplementary Information

Below is the link to the electronic supplementary material.

Supplementary file1 (DOCX 294 KB)
