# Supplementary material for: Ethics at the Intersection of Intelligent Assistive Technology, Ageing, and the Home Environment: A Scoping Review
Source: J Bioeth Inq. 2025 Oct 6;23(2):389–403. doi: 10.1007/s11673-025-10479-8 (PMC13388638; doi:10.1007/s11673-025-10479-8)
Supplement: Supplementary file 1 — Supplementary file1 (DOCX 294 KB) [file 11673_2025_10479_MOESM1_ESM.docx]

Table 1: Full list of search terms in five keyword categories and exemplary search string for WebofScience.

| Category | Search terms |
| --- | --- |
| Older age | ‘aged’, ‘elder*’, ‘older*’, ‘ag*ng’, ‘senior*’, ‘geriatr*’, ‘gerontolo*’, ‘later life’ |
| Home environment | ‘ag*ing at home’, ‘ag*ing in place’, ‘community dwelling*’, ‘independent living’, ‘assisted living’ |
| Technology | ‘telecare’, ‘gerontechn*’, ‘welfare techn*’, ‘smart home’, ‘smart house’, ‘intelligent assistive technolog*’, ‘mobile health’, ‘digital technolog*’, ‘smart techno*’, ‘smart living’, ‘intelligent living’, ‘ambient assistive living’, ‘assistive technolg*’, ‘smart ag*ing’, ‘wearab*’, ‘intelligent assistive technolog*’ |
| Health | ‘health*’, ‘prevention’, ‘healthy ag*ing’, ‘well-being’ |
| Ethics | ‘ethic*’, ‘autonom*’, ‘quality of life’, ‘ag*ing well’, ‘moral*’, ‘normative’, ‘value’, ‘evaluative’, ‘bioethic*’, ‘digital ethic*’ |
| Search string (example WebofScience) | TS=(“aged” OR “elder*” OR “older*” OR “ag*ng” OR “senior*” OR “geriatr*” OR “gerontolo*” OR “later life”) AND TS=(“ag*ing at home” OR “ag*ing in place” OR “community dwelling” OR “independent living” OR “assisted living”) AND TS=(“telecare” OR “gerontechn*” OR “welfare techn*” OR “smart home” OR “smart house” OR “intelligent assistive technolog*” OR “mobile health” OR “digital technolog*” OR “smart techno*” OR “smart living” OR “intelligent living” OR “ambient assistive living” OR “assistive technologies” OR “smart ag*ing” OR “wearab*”) AND TS=(“health*” OR “prevention” OR “healthy ag*ing” OR “well-being”) AND TS=(“ethic*” OR “autonom*” OR “quality of life” OR “ag*ing well” OR “moral*” OR “normative” OR “value” OR “evaluative” OR “bioethic*” OR “digital ethic*”) |

*Table 2: Assignment of ethical aspects from the literature according to principlism.*

| **Respect for Autonomy** | **Decision** | willingness to use the technology (Sánchez, Taylor, and Bing-Jonsson 2017)  the decision of who gets access to the data (Chung, Demiris, and Thompson 2016; Zhu et al. 2021)  being in control of the decision (Sánchez et al. 2019; Ehrari, Ulrich, and Andersen 2020)  the right to accept or reject a technological system (Zhu et al. 2021)  making own decisions without interfering with third parties (Schicktanz and Schweda 2021; Panico et al. 2020; Sánchez et al. 2019)  who decides to use certain technologies – older adult/family/caregiver (Zwijsen, Niemeijer, and Hertogh 2011; Zhu et al. 2021; Schicktanz and Schweda 2021; Sánchez et al. 2019; Sonnauer and Frewer 2023)  having control over the technology (Pirzada et al. 2022; Sundgren, Stolt, and Suhonen 2020; Zhu et al. 2021; Felber et al. 2023)  willingness to share data to increased autonomy through improved healthcare outcomes (Ehrari, Ulrich, and Andersen 2020)  being aware that decisions are often influenced by interdependent relationships, different older adults may have different needs and contexts for decision-making (McLean 2011)  decisions made by researchers about system design and the choices given to end-users (Birchley et al. 2017) | |
| --- | --- | --- | --- |
|  |  | **Informed Consent** | informed decision-making (Chung, Demiris, and Thompson 2016)  a sign of willingness (Sánchez, Taylor, and Bing-Jonsson 2017)  informed consent and the ability to make choices (Zhu et al. 2021)  informed consent as an instrument to ensure autonomy (Zwijsen, Niemeijer, and Hertogh 2011)  informed consent should be regarded as an ongoing process and not a singular event (Sánchez, Taylor, and Bing-Jonsson 2017; Hartmann, Primc, and Rubeis 2023)  informed consent as an instrument of overcoming ethical challenges – privacy concerns (Sánchez, Taylor, and Bing-Jonsson 2017)  challenges to give informed consent without knowing (missing knowledge) about the technology/no experience with the technology (Zwijsen, Niemeijer, and Hertogh 2011)  possibility to give informed consent is often influenced by too simple or too difficult information – which makes it difficult to make an informed decision (Zhu et al. 2021)  older adults often do not have much interest in the functionality of technology but rather in its purpose – therefore, healthcare personnel have a special role in conveying knowledge and information about technology to encourage informed decision-making (Chung, Demiris, and Thompson 2016) |
|  | **Control** | who controls the technology when the capacity of the respective user to do so declines (Sánchez et al. 2019)  having control over the data (Chung, Demiris, and Thompson 2016; Zhu et al. 2021; Felber et al. 2023; Ji and Kim 2022; Ehrari, Ulrich, and Andersen 2020; Hartmann, Primc, and Rubeis 2023)  control over technology, for example, interaction with the system, turn on and off; when and how it is used (Zhu et al. 2021; Felber et al. 2023; Hartmann, Primc, and Rubeis 2023)  family and caregivers pressuring older adults to use certain technology (Sundgren, Stolt, and Suhonen 2020; Zwijsen, Niemeijer, and Hertogh 2011)  pressure to use technology (Sundgren, Stolt, and Suhonen 2020; Zwijsen, Niemeijer, and Hertogh 2011; Felber et al. 2023)  pressure: feeling frail and dependent through the pressure from family to use certain technologies (Pirzada et al. 2022)  protecting a personal way of life (Schicktanz and Schweda 2021; Sonnauer and Frewer 2023)  pressure to use technologies in specific ways to avoid alarms or emergency responses can lead to standardized behaviour, limiting users’ flexibility and autonomy (Hartmann, Primc, and Rubeis 2023; Birchley et al. 2017)  the need to protect the autonomy and dignity of older adults by avoiding practices that may be disrespectful or demeaning, such as deception, infantilization, or paternalism (Felber et al. 2023)  fear of losing autonomy due to excessive monitoring or dependence on technology (Felber et al. 2023; Sonnauer and Frewer 2023)  limitation of user’s choice due to technical constraints (Ji and Kim 2022; Birchley et al. 2017)  control should not be equated with autonomy as this can become harmful and lead to an interpretation of failure (McLean 2011)  end-user choice interpreted as a key strategy to solve ethical dilemmas, especially around privacy and consent (Birchley et al. 2017) | |
|  | **Privacy** | confidentiality (Chung, Demiris, and Thompson 2016)  a state where one is not observed or disturbed by others (Zwijsen, Niemeijer, and Hertogh 2011)  increased privacy in comparison to moving to a nursing home (Sundgren, Stolt, and Suhonen 2020; Ehrari, Ulrich, and Andersen 2020; Ji and Kim 2022; Felber et al. 2023)  having more privacy through the use of technology than being cared for by relatives (Sánchez, Taylor, and Bing-Jonsson 2017)  pressure to accept data tracking to maintain independent living (Hartmann, Primc, and Rubeis 2023)  concerns about how data is collected, stored, shared, and protected (Birchley et al. 2017; McLean 2011)  monitoring devices can be intrusive or overly restrictive, potentially compromising an individual's sense of independence (Bennett 2019; Ji and Kim 2022)  strong focus on informational privacy (e.g., data protection) rather than physical privacy (e.g., avoiding intrusive monitoring) (Birchley et al. 2017)  challenge of balancing user autonomy with privacy protection (Birchley et al. 2017; Bennett 2019; Ji and Kim 2022; Ehrari, Ulrich, and Andersen 2020) | |
|  | **Interdependence** | autonomy is dependent and influenced by a web of social relationships (McLean 2011; Sonnauer and Frewer 2023)  needing help should not be interpreted as a lack autonomy (McLean 2011)  self-determination is not dependent on independence, as a person can be dependent on external support but still maintain self-determent and vice versa (Hartmann, Primc, and Rubeis 2023)  concept of independence often centers on activities of daily living, while pragmatically useful, this can reduce autonomy by promoting a normative view of independence that overlooks social determinants and individual diversity (Hartmann, Primc, and Rubeis 2023) | |
| **Beneficence** | **Safety** | being safe (Sánchez et al. 2019; Ienca et al. 2017)  getting support when needing it (Mortenson, Sixsmith, and Beringer 2016; Ehrari, Ulrich, and Andersen 2020; Bennett 2019; Sundgren, Stolt, and Suhonen 2020)  Ambient assisted living would make them feel more secure (Mortenson, Sixsmith, and Beringer 2016)  preparedness for unpredictable scenarios (Sánchez et al. 2019)  many older adults said that they would buy any device as long as it makes life easier and supports being safe and affordable (Sánchez et al. 2019)  safety as a powerful driver for acceptance (Zhu et al. 2021; Sundgren, Stolt, and Suhonen 2020; Ienca et al. 2017) | |
|  | **Relationships** | Assistive technology could lead to more social contact over video calling, etc. (Zwijsen, Niemeijer, and Hertogh 2011)  scientists see the technology as a supplement for contact (Zhu et al. 2021)  an important addition to face-to-face contact additionally concerning the shortage of nurses and human health resources (Zhu et al. 2021; Bennett 2019)  creating conditions that empower individuals to retain autonomy while receiving the benefits of technology (Sonnauer and Frewer 2023) | |
|  | **Empowerment** | **Living at home** | being able to live at home (Sánchez et al. 2019; Ehrari, Ulrich, and Andersen 2020)  possibility to age in place and age independently (Pirzada et al. 2022)  rise in self-esteem and self-confidence through being able to stay at home (Sundgren, Stolt, and Suhonen 2020)  when not feeling safe everything is tried to make it better – increase the use of technology with the aim of staying at home as long as possible (Ienca et al. 2017)  to support the individual’s residual physical and cognitive ability (Panico et al. 2020)  peace of mind for relatives and informal carers (Sundgren, Stolt, and Suhonen 2020)  emotional relief for carers (Zhu et al. 2021)  reducing the burden on family and caregivers (Pirzada et al. 2022) |
| **Non-Maleficence** | **Safety** | technology fails to detect emergencies, such as falls (Sánchez, Taylor, and Bing-Jonsson 2017)  technical errors and inaccurate measurements can cause harm (Sánchez, Taylor, and Bing-Jonsson 2017; Moraitou, Pateli, and Fotiou 2017; Schicktanz and Schweda 2021; Panico et al. 2020)  need to proactively identify and mitigate risks in technology development (McLean 2011)  ensuring safety and, at the same time, protecting personal privacy (Bennett 2019)  sense of security through enthusiasm for the technology expressed by the family (Ehrari, Ulrich, and Andersen 2020) | |
|  | **Privacy** | **Misuse of Data** | risk of misuse of the data, for example, through insurance (Ienca et al. 2017; Birchley et al. 2017; McLean 2011; Felber et al. 2023; Ehrari, Ulrich, and Andersen 2020)  abuse of information or used for stigmatization (Zhu et al. 2021)  sharing information without permission and obtaining information without one’s will (Chung, Demiris, and Thompson 2016)  commercialization as a potential problem (Flick et al. 2020)  invasion of personal space or the misuse of personal information (McLean 2011; Ji and Kim 2022) |
|  |  | **Surveillance** | camera-based surveillance is a major risk to privacy (Ienca et al. 2017; Sonnauer and Frewer 2023; Hartmann, Primc, and Rubeis 2023)  use of cameras is understood as a major risk for privacy violation (Zwijsen, Niemeijer, and Hertogh 2011; Hartmann, Primc, and Rubeis 2023)  Ambient assisted living as a panopticon (Mortenson, Sixsmith, and Beringer 2016)  monitoring is understood as a loss of dignity in a person’s own home (Pirzada et al. 2022)  surveillance could lead to self-surveillance (Mortenson, Sixsmith, and Beringer 2016; Hartmann, Primc, and Rubeis 2023; Rubeis 2020) |
|  |  | **Obstrusiveness** | perception of privacy invasion is related to the extent of detailed data the technology collects, for example, motion detection compared to sound or image capturing (Chung, Demiris, and Thompson 2016; Bennett 2019)  feeling disturbed by the technology, depending on respective technology and installation location, can often trigger anxieties (Zwijsen, Niemeijer, and Hertogh 2011; Felber et al. 2023)  associated with installation location – bedroom, bathroom, and types and sizes of technology (Chung, Demiris, and Thompson 2016)  some think that the use of cameras would be inappropriate (Sánchez et al. 2019)  installing a video camera is seen as a major privacy violation (Chung, Demiris, and Thompson 2016)  noises of flickering lights become a nuisance or could trigger anxiety (Chung, Demiris, and Thompson 2016)  blurring private space with public space through the use of technology could lead to losing control over private space which, in turn, could lead to reduced self-esteem (Zhu et al. 2021) |
|  | **Relationships** | fewer visits: for example, caregivers stop by less because of data (Zwijsen, Niemeijer, and Hertogh 2011; McLean 2011)  reduced human contact (Zhu et al. 2021; McLean 2011)  losing face-to-face contact could lead to depression/isolation (Flick et al. 2020)  Assistive technology use might lead to loss of human contact; isolation (Zwijsen, Niemeijer, and Hertogh 2011; Flick et al. 2020)  experience that smart homes restrict users from social interactions (Pirzada et al. 2022)  risk of lack of human touch (Moraitou, Pateli, and Fotiou 2017; Felber et al. 2023; Ienca et al. 2017)  risk of reduced social contact and isolation (Sánchez, Taylor, and Bing-Jonsson 2017; McLean 2011)  risk of reduced communication with social networks or health professionals; risk of isolation and reduced well-being (Sánchez, Taylor, and Bing-Jonsson 2017)  technology may increase the feeling of being safe only at home with the risk of isolation (Pirzada et al. 2022)  telecare could lead to discouraging older people from maintaining personal contact and have effects on their mobility and general well-being (Sundgren, Stolt, and Suhonen 2020)  fear of extra work time due to integrating technologies into care (Felber et al. 2023)  depersonalization of care through the use of AI applications (Rubeis 2020) | |
|  | **Stigma** | increased self-image of being disabled through using IAT (Panico et al. 2020; McLean 2011; Felber et al. 2023)  labeling those who need such devices for safety reasons can lead to stigma (Mortenson, Sixsmith, and Beringer 2016)  stigma and ageism as reasons why older adults do not adapt to technology – especially when they are told that this specific technology is for frail, vulnerable older people (Pirzada et al. 2022)  embarrassed when a device would make a noise in public (Zwijsen, Niemeijer, and Hertogh 2011)  using technology is a sign of frailty and dependence and is, therefore, stigmatizing (Zwijsen, Niemeijer, and Hertogh 2011; Felber et al. 2023)  change of perceptions of the users of IAT; highlighting disabilities (Sánchez, Taylor, and Bing-Jonsson 2017; Rubeis 2020)  bias from the side of the developers: perpetuates prejudices and leads to exclusion; older adults not being seen as people but rather as diseases/disabilities (Flick et al. 2020)  older adults were concerned about the potential judgment of their activity patterns through sensor data (Chung, Demiris, and Thompson 2016)  objectifying the user; mainly focusing on data (Flick et al. 2020)  the shame of not knowing how to use technology; feeling incompetent to use a device, despite the desire to use it; push-button alarms as a reminder that you are old (Flick et al. 2020)  concerned about the ability to learn how to use new technology (Sundgren, Stolt, and Suhonen 2020; Felber et al. 2023)  technology as a reminder that you have a serious problem for which you need ambient assisted living (Mortenson, Sixsmith, and Beringer 2016)  those who already use the technology bought it so as not to be a burden (Sánchez et al. 2019)  age scripts as stereotypical concepts inscribed in technologies (Rubeis 2020; Rubeis, Fang, and Sixsmith 2022) | |
|  | **Sustainability** | sustainability, sense of scarce resources, low waste, and renewable energy (Schicktanz and Schweda 2021) | |
| **Justice** | **Distribution of Healthcare** | **Accessibility** | barriers to the use of the technology, such as economic, usability, or language; and economic gap between users; costs and affordability (Zhu et al. 2021; Chung, Demiris, and Thompson 2016; Rubeis, Fang, and Sixsmith 2022; Ji and Kim 2022; Bennett 2019)  cost-effective for users and the healthcare system (Sánchez, Taylor, and Bing-Jonsson 2017)  costs of technology (Chung, Demiris, and Thompson 2016; Sánchez, Taylor, and Bing-Jonsson 2017; Sánchez et al. 2019)  who can afford and use technology (McLean 2011; Pirzada et al. 2022)  usability of critical importance for accessibility for the users; designers should be aware of age-related limitations (Moraitou, Pateli, and Fotiou 2017)  concerns about the stable functioning of the technology (Sundgren, Stolt, and Suhonen 2020)  people who could benefit the most from AgeTech experience the greatest access barriers due to social determinants and technology deterrents (Rubeis, Fang, and Sixsmith 2022)  diversifying training data, models, and algorithms – algorithmic fairness (Rubeis, Fang, and Sixsmith 2022) |
|  |  | **Cost Coverage** | who pays for the technology (Pirzada et al. 2022)  what is financed to guarantee care for older adults (Schicktanz and Schweda 2021)  acceptance of technology increasses when children or the government covers the costs: the technologies supporting well-being should either be made less expensive or subsidized for a more inclusive society (Pirzada et al. 2022)  when assistive technologies are rated as care services, they might be reimbursed by healthcare insurance (Zwijsen, Niemeijer, and Hertogh 2011) |
